# A Comprehensive Analysis of Renal and Endothelium Dysfunction Markers Fourteen Years after Hemorrhagic Fever with Renal Syndrome Contraction

**DOI:** 10.3390/life14050575

**Published:** 2024-04-30

**Authors:** Dragan Ledina, Ivo Ivić, Ante Tadin, Kristian Bodulić, James W. LeDuc, Alemka Markotić

**Affiliations:** 1 Department of Infectious Diseases, Split University Hospital, 21000 Split, Croatia; dragan.ledina@yahoo.com (D.L.); ivo@radogost.com (I.I.); 2 School of Medicine, University of Split, 21000 Split, Croatia; 3 Research Department, University Hospital for Infectious Diseases “Dr. Fran Mihaljević”, 10000 Zagreb, Croatia; ante.tadin@yahoo.com (A.T.); kbodulic@bfm.hr (K.B.); 4 Department of Microbiology and Immunology, University of Texas Medical Branch, Galveston, TX 77550, USA; jwleduc@utmb.edu; 5 School of Medicine, Catholic University of Croatia, 10000 Zagreb, Croatia; 6 Faculty of Medicine, University of Rijeka, 51000 Rijeka, Croatia

**Keywords:** HFRS, Puumala virus, Dobrava virus, renal dysfunction, endothelial dysfunction

## Abstract

While the pathology of acute hemorrhagic fever with renal syndrome (HFRS) has been widely researched, details on the chronic HFRS sequelae remain mainly unexplored. In this study, we analyzed the clinical and laboratory characteristics of 30 convalescent HFRS patients 14 years after the disease contraction, mainly emphasizing several endothelial dysfunction parameters. Convalescent HFRS patients exhibited significantly higher serum levels of erythrocyte sedimentation rate, von Willebrand factor, uric acid, C-reactive protein and immunoglobulin A when compared to healthy individuals. Furthermore, 24 h urine analyses revealed significantly lower sodium and potassium urine levels, as well as significantly higher proteinuria, microalbumin levels and β2-microglobulin levels when compared to healthy individuals. First morning urine analysis revealed significantly higher levels of hematuria in convalescent HFRS patients. None of the additional analyzed endothelium dysfunction markers were significantly different in post-HFRS patients and healthy individuals, including serum and urine P-selectin, E-selectin, soluble intercellular adhesion molecule 1, vascular intercellular adhesion molecule 1 (sVCAM-1) and vascular endothelial growth factor (VEGF). However, binary logistic regression revealed a weak association of serum sVCAM-1 and urine VEGF levels with HFRS contraction. Generally, our findings suggest mild chronic inflammation and renal dysfunction levels in convalescent HFRS patients 14 years after the disease contraction.

## 1. Introduction

Hemorrhagic fever with renal syndrome (HFRS) is a zoonotic disease caused by the *Orthohantavirus* genus (hantaviruses, HTV). Two pathogenic HTVs, Puumala virus (PUUV) and Dobrava virus (DOBV), are the main causative agents of HFRS in Europe [1,2,3,4,5,6]. The virology, epidemiology and clinical features of acute HFRS are well documented, but little is known about the chronic sequelae of HFRS and the accompanying immunopathogenesis. Acute HFRS patients usually exhibit various symptoms, including fever, strong headache, myalgia, back pain and nausea. However, severe cases can result in manifestations such as blurred vision, pneumonitis and bleeding [1,2,3,4,6]. These symptoms can be followed by different degrees of renal involvement, possibly manifesting in oliguria, hematuria and proteinuria [1,2,3,4,6]. The disease course is usually accompanied by pathological laboratory findings, including leukopenia and thrombocytopenia, as well as C-reactive protein (CRP), urea, creatinine and liver transaminase elevation [3,4,5,6].

Endothelial cells (ECs), monocytes and macrophages represent the main target cells of HTV. The main pathophysiological changes in infected ECs include increased vascular permeability accompanied by endothelial activation, increased expression of adhesion molecules and recruitment of inflammatory cells in the involved organs. In HFRS pathology, vascular cells are both a target of cytokines and chemokines, as well as a source of their production [7,8]. The majority of pathophysiological changes during HFRS occur in the kidneys. As such, renal endothelium dysfunction (ED) plays a key role in the development and maintenance of HFRS-associated renal vascular permeability [3,4,5,6,9,10]. Several studies demonstrated a negative association between glomerular filtration rate (GFR) and renal inflammatory activity during HFRS [11,12]. Moreover, evidence suggests a significant elevation of several acute kidney injury (AKI) markers in HFRS patients [4,5,9,13]. Endothelial damage in patients with AKI during HFRS results in elevated markers of ED [7,8]. Typically, ED detection is based on measuring the levels of circulating markers of endothelial function such as endothelin-1, tissue plasminogen activator (tPA), plasminogen activator inhibitor (PAI) and vascular endothelial growth factor (VEGF). Additionally, ED detection can be established by measuring the levels of adhesion molecules, such as soluble intercellular adhesion molecule 1 (sICAM-1), soluble vascular cell adhesion molecule 1 (sVCAM-1), P-selectin and E-selectin [14,15,16]. Notably, several studies found increased levels of VEGF in the sera and urine of HFRS patients [17]. Similarly, recent evidence suggests increased levels of endothelin-1, tPA, PAI, sICAM-1, sVCAM-1 and selectins in the sera of HFRS patients [13,18,19,20]. However, data on urine levels of endothelin-1, sICAM-1, sVCAM-1 and selectins during HFRS are still lacking, with only one study suggesting non-pathological levels of endothelin-1 in Croatian HFRS patients [13].

While the pathology of HFRS has been extensively researched, little is known about chronic HFRS sequelae. So far, very few follow-up studies related to chronic HFRS after PUUV or DOBV infection have been published. Three of the stated studies suggested tubulointerstitial changes, persistently lower levels of GFR and higher levels of proteinuria, hematuria and other AKI markers three, six and ten years after acute HFRS [21,22,23]. Similarly, a study conducted by Dudarev et al. emphasized the importance of glomerular and tubular dysfunction in convalescent HFRS [24]. On the contrary, a recent study reported physiologically normal GFR values in HFRS patients ten years after PUUV or DOBV infection [11]. Notably, none of the stated research studied HFRS sequelae in the context of renal ED. In this study, we investigate the existence and severity of chronic renal sequelae, as well as their association with ED markers 14 years after HFRS contraction in patients infected with PUUV or DOBV during an HFRS outbreak in Croatia.

## 2. Materials and Methods

This study recruited 30 adult HFRS patients hospitalized in Split University Hospital (Croatia) during the HFRS epidemic in 1995. All patients were prospectively analyzed 14 years after PUUV or DOBV infection. Patient exclusion criteria included malignant disease, epilepsy, diabetes, cerebrovascular insult, myocardial infarction, permanent dialysis and treatment with angiotensin convertase inhibitors, Ca^2+^ blockers or angiotensin II receptor antagonists. Similarly, 30 age- and sex-matched healthy individuals were analyzed during the corresponding time period. Exclusion criteria for the control group were as follows: chronic diseases (including chronic renal disease and hypertension) and treatment with angiotensin convertase inhibitors, Ca^2+^ blockers or angiotensin II receptor antagonists. Both the patient and control groups were tested for PUUV and DUBV infection with ELISA tests for specific immunoglobulin G (IgG) antibodies. All participants signed an informed consent form. This study was conducted in accordance with the Declaration of Helsinki and was approved by the Ethics Committee of the School of Medicine, University of Zagreb.

Complete anamnesis was taken for all of the analyzed patients. All participants underwent a complete physical examination. Three blood pressure measurements in the seated position within time intervals of 30 min were taken and subsequently averaged for data analysis. This included systolic pressure, diastolic pressure and mean arterial pressure calculated with the following formula: 1/3 * (systolic pressure—diastolic pressure) + diastolic pressure. Abdominal and liver ultrasounds were performed on all participants (Toshiba “Nemio” SSA-550 A, Tokyo, Japan). Participants’ sera were collected after physical examination. Standard hematological parameters were measured on a hematology analyzer (Advia 120 Hematology System Bayer Corporation, Tarrytown, New York, NY, USA) and included erythrocyte sedimentation rate (ESR), erythrocyte count, hemoglobin, leukocyte count and platelet count. Furthermore, von Willebrand factor (vWF) and D-dimers were measured on a suitable analyzer (Behring Coagulation Timer, Version 1.7, Dade Behring Gmbh, Marburg, Germany). The biochemical analysis included the assessment of aspartate aminotransferase (AST), urea, uric acid, triiodothyronine (T3), thyroxin (T4), CRP and immunoglobulin A (IgA) levels. Regarding urine analysis, three consecutive samples of 24 h urine were collected following a three-day NaCl-restricted diet (≤6 g/day). The measured values were averaged across the three collected urine samples for data analysis. This analysis encompassed electrolyte (sodium, potassium, chloride), urea, urine protein, microalbumin and β2-microglobulin level determination (Olympus AU 2700, Olympus Mishima Co., Ltd., Shizuoka, Japan). Additionally, GFR was calculated using Lavy’s formula. The first morning urine samples were cultured and analyzed for nitrites, urine proteins, erythrocytes and leukocytes. Specific ED markers were measured in serum and urine samples, including P-selectin, E-selectin, sICAM-1, sVCAM-1 and VEGF, using ELISA tests (Quantikine, R&D System, Oxford, UK).

Quantitative variables are expressed as medians and ranges, while qualitative variables are given as absolute values and percentages. Distribution normality was assessed graphically and with the Shapiro–Wilk test. Quantitative variables with nonparametric distributions were compared using the Mann–Whitney test. Association between categorical variables was assessed using the chi-square or Fisher’s exact test, as appropriate. The relationship between numeric variables was assessed using Spearman’s correlation coefficient and the correlation test. Binary logistic regression was utilized to classify participants into post-HFRS patients and healthy individuals, with predictor sets chosen using the forward selection algorithm. The model was evaluated on the training dataset using specificity and selectivity measures. All tests were two-tailed with the significance factor set to 95%. Statistical analyses were performed using R (version 4.1.2.) [25].

## 3. Results

### 3.1. General Participant Characteristics

This study recruited 30 post-HFRS patients and 30 age- and sex-matched healthy individuals. Participants’ demographic and clinical parameters are shown in Table 1. All patients were male with a median age of 41 years (range 31–49 years) and a median body weight of 89 kg (range 61–111 kg). Furthermore, 90% of patients were infected with PUUV, 6.7% of patients were infected with DOBV and the viral species of one patient was undetermined due to cross-reactivity. We did not observe a significant difference between the patients and the control group in terms of age or body weight (*p* > 0.05). The most common post-HFRS symptoms included headache (50.0%), depression (50.0%), painful lumbar succussion (36.7%), hepatomegaly (10,0%), frequent urination (6.7%) and splenomegaly (6.7%). When compared to healthy individuals, we found significantly higher systolic pressure (medians 130.0 and 123.3 mmHg, *p* = 0.025), diastolic pressure (medians 86.7 and 80 mmHg, *p* = 0.001) and mean arterial pressure (medians 101.1 and 91.6 mmHg, *p* < 0.001) levels in post-HFRS patients.

### 3.2. Hematological and Biochemical Parameters in Post-HFRS Patients

Firstly, we analyzed the potential differences in post-HFRS patients and healthy individuals in the selected hematological and biochemical parameters (Table 2). When compared to healthy individuals, post-HFRS patients exhibited significantly higher ESR (medians 6 and 4 mm/h, *p* = 0.025) and vWF levels (medians 1.24 and 1.04 IU/L, *p* = 0.008). On the other hand, we did not find a significant difference in post-HFRS patients and the control group in hemoglobin levels, D-dimer levels and erythrocyte, leukocyte or platelet count (*p* > 0.05). Furthermore, post-HFRS patients exhibited significantly increased levels of uric acid (medians 319 and 297 µmol/L, *p* = 0.025), CRP (medians 2.5 and 1.2 mg/L) and IgA (medians 2.3 and 1.8 g/L, *p* = 0.014), as well as decreased levels of T4 (medians 87 and 104 mmol/L, *p* = 0.005). We did not find a significant difference between the serum levels of AST, urea or T3 (*p* > 0.05). Notably, medians of all of the stated parameters were within the reference value range of the respective parameters for both the post-HFRS patient group and the control group.

### 3.3. Urine Tests in Post-HFRS Patients

In the following part of the study, we compared the results of 24 h urine tests in post-HFRS patients and healthy individuals (Table 3). When compared to healthy individuals, post-HFRS patients exhibited significantly lower urine levels of sodium (medians 157 and 230 mmol/d, *p* = 0.040) and potassium (medians 62 and 65 mmol/d, *p* = 0.010). We also noticed lower levels of chlorides (medians 185 and 223 mmol/d), which were not statistically significant (*p* = 0.061). Several urine parameters were significantly higher in post-HFRS patients than in the control group. These parameters included urine proteins (medians 133 and 90 mg/d, *p* = 0.023), microalbumin (medians 6.9 and 5.3 mg/d, *p* = 0.011) and β2-microglobulin (medians 0.20 amd 0.10 mg/L, *p* = 0.027). Again, medians of all of the 24 h urine parameters were within the reference interval of the respective parameters for both the post-HFRS patient group and the control group.

We also analyzed the first morning urines of post-HFRS patients and the control group (Table 4). We did not record statistically significant differences in urine-positive nitrite, proteinuria or leukocyturia frequencies (*p* > 0.05). However, post-HFRS patients exhibited significantly higher frequencies of hematuria compared to healthy individuals (OR = 5.07, 95% CI 1.13–31.12, *p* = 0.030). Furthermore, we did not find a significant difference in the GFR of post-HFRS patients and healthy individuals (medians 88.00 and 87.39 mL/min, *p* > 0.05). Finally, post-HFRS patients displayed significantly lower morning urine osmolality when compared to healthy individuals (medians 830 and 880 mOsm/kg H20, *p* = 0.011).

### 3.4. Specific Endothelial Dysfunction Markers in Post-HFRS Patients

Finally, we examined specific ED parameters in serum and 24 h urine samples of post-HFRS patients (Table 5). While several serum ED markers exhibited higher values in post-HFRS patients than in healthy individuals, none of the differences were statistically significant (P-selectin: medians 101.4 and 84.0 ng/mL; E-selectin: medians 50.3 and 46.3 ng/mL; sICAM-1: medians 205.2 and 165.3 ng/mL; and VEGF: 278.5 and 236.0 pg/mL, *p* > 0.05). When considering urine ED markers, post-HFRS patients exhibited higher sVCAM-1 (medians 20.6 and 5.3 ng/mL) and VEGF (medians 276.1 and 242.2 pg/mL) levels. However, these differences were not statistically significant (*p* = 0.080 and *p* = 0.294, respectively). The urine levels of other specific ED markers were similar in the post-HFRS patient and control groups (P-selectin: medians 0.1 and 0.1 ng/mL; E-selectin: medians 0.2 and 0.2 ng/mL; and sICAM-1: medians 0.0 and 0.0 ng/mL, *p* > 0.05). 

We also evaluated the potential correlation of hematological, biochemical and 24 h urine parameters with specific serum and urine ED markers. We found a moderate positive correlation between urine microalbumin and serum sVCAM-1 levels (r = 0.45, *p* = 0.044) and between E-selectin and VEGF serum levels (r = 0.40, *p* = 0.030).

### 3.5. Multivariate Analysis of Post-HFRS Patient Markers

Finally, we assessed the potential of the analyzed laboratory markers in classifying post-HFRS patients and healthy individuals using binary logistic regression (Table 6). Analyzed parameters with the best predictive ability for post-HFRS were serum CRP, 24 h urine microalbumin, serum sVCAM-1 and urine VEGF levels. Increasing the levels of serum CRP by one unit would increase the odds of a participant being in the post-HFRS patient group by 2.691 (*p* = 0.050). Furthermore, increasing the levels of 24 h urine microalbumin would increase the odds of a participant being in the post-HFRS patient group by 1.474 (*p* = 0.048). Finally, increasing the levels of urine VEGF by one unit would increase the odds of a participant being in the post-HFRS patient group by 1.015 (*p* = 0.041). The accuracy of the stated model on the training dataset was 91.1% (sensitivity: 93.1%; specificity: 88.9%).

## 4. Discussion

This prospective study aimed to reduce the research gap in HFRS sequelae and the potential impact of HFRS on long-term patient health. The clinical course of acute HFRS is manifested by immunopathological events and ED, often leading to vascular leakage and hypertension [1,2,3,4,5,6]. One of the main consequences of renal ED and local inflammation during acute HFRS is AKI, possibly resulting in manifestations such as lower GFR, proteinuria, leukocyturia, hematuria and, in severe cases, renal failure [4,5,6,9]. On the other hand, the dynamics of chronic changes following acute HFRS are partially known. Endothelium impairment in acute HFRS could, in some patients, potentially induce chronic changes in microcirculation, leading to post-HFRS events such as hypertension, permanent damage of small blood vessels in glomeruli and the development of microalbuminuria [10,11].

In this study, we performed detailed hematological, biochemical and urine analyses in post-HFRS patients 14 years after HFRS contraction. To our knowledge, this represents the longest time period of HFRS patients monitoring in the published research [11,21,22,23,24]. Our results reveal significant changes in several markers of chronic inflammation and renal dysfunction. Several studies reported significantly higher values of ERS and serum CRP levels during acute HFRS, as well as their correlation with HFRS-related systemic and local inflammation [5,26]. In this context, the slightly elevated ESR and CRP levels found in our study could indicate chronic inflammatory processes in post-HFRS patients. This represents an important result considering that inflammatory markers have not been extensively studied in HFRS sequelae. Importantly, our results also indicate significantly higher levels of vWF in post-HRFS patients, possibly pointing toward chronic ED. When considering other hematological parameters, we did not observe a significant difference in erythrocyte count or hemoglobin levels between post-HFRS patients and healthy individuals. While anemia represents a common laboratory finding during acute HFRS [27], our findings indicate normal erythrocyte levels in post-HFRS patients. Interestingly, the post-HFRS patients analyzed in this study exhibited significantly higher levels of serum uric acid, possibly resulting from lower renal function or hypertension. This finding agrees with the results of a study conducted by Dodarev et al., hypothesizing glomerular and tubular dysfunction in post-HFRS patients as a result of glomerular hypertension and tubular damage [24]. The finding of lower T4 levels in post-HFRS patients could be a result of chronic inflammation and leukocytosis, agreeing with trends commonly observed in acute HFRS [28]. When considering liver function, the levels of AST were not significantly different in post-HFRS patients, with a relatively low percentage of patients exhibiting hepatomegaly or other ultrasound abnormalities. All in all, hematological and biochemical analyses conducted in our study could point towards relatively mild levels of chronic inflammation in convalescent HFRS. 

The results of 24 h urine tests in post-HFRS patients revealed significantly lower levels of sodium and potassium, as well as significantly higher levels of urine proteins, microalbumin and β2-microglobulin. The lower levels of urine electrolytes observed in the studied patients could suggest a lower tubular reabsorption capacity, agreeing with the results of a similar study on post-HFRS patients [24]. Our results also demonstrate significantly higher proteinuria and microalbuminuria levels when analyzing 24 h urines of post-HFRS patients, as well as a higher frequency of proteinuria in the morning urine of post-HFRS patients, which was not statistically significant. The presented findings agree with the results of similar studies on convalescent HFRS [11,22,23] and contextualize proteinuria as an important renal dysfunction marker persisting for 14 years after acute HFRS. While the pathological mechanism of mild proteinuria in convalescent HFRS is not known, this finding points towards glomerular filtration disturbance possibly caused by hypertension and renal vasculature damage. Our results also reveal significantly higher 24 h urine β2-microglobulin levels and a positive correlation between serum CRP levels, proteinuria and β2-microglobulin. These results are in line with the observations of other studies on HFRS sequelae and may suggest post-HFRS renal tubule damage as a consequence of local chronic inflammation [11,22,23]. However, it should be noted that the medians of the majority of hematological and urine test parameters of the analyzed patients corresponded to reference values. Consequently, definite conclusions on chronic inflammation in convalescent HFRS cannot be drawn. Several of the analyzed post-HFRS patients also exhibited hematuria as a sign of glomerular damage, coinciding with the results of the study conducted by Sirotin et al. [21]. The analyzed patients also had lower osmolality, agreeing with the findings of a similar study and implying urine concentration defects [28]. Importantly, the patients analyzed in our study did not exhibit significantly lower levels of GFR when compared to the healthy control group. Considering that similar studies conducted three and six years after acute HFRS reported lower GFR levels, this finding could imply the eventual normalization of kidney function in later post-HFRS time points. Taken together, the results of the urine tests conducted in our study suggest milder levels of renal tubule damage as a possible consequence of hypertension and local inflammation 14 years after acute HFRS onset. 

A significant part of our study consisted of analyzing additional ED markers in post-HFRS patients’ sera and urine. When considering acute HFRS, several studies demonstrated increased levels of several markers, including sICAM-1, sVCAM-1 and selectins [18,19]. However, ED markers in convalescent HFRS have not been analyzed, highlighting the importance of our study. While several serum ED markers, including P-selectin, E-selectin, sICAM-1 and VEGF, were increased in post-HFRS patients, these differences were not statistically significant when compared to healthy individuals. Similarly, urine sVCAM-1 values were on average fourfold higher in post-HFRS patients without reaching a statistically significant difference when compared to the control group, possibly due to data dispersion and a relatively small sample size. Consequently, concluding on the basis of these results should be carried out with caution. Furthermore, our logistic regression model demonstrated a weak association of serum levels of sVCAM-1 and urine levels of VEGF with HFRS contraction. Notably, this model achieved considerably high sensitivity and specificity in classifying post-HFRS patients and healthy individuals using only four predictors: CRP, urine microalbumin, serum sVCAM-1 and urine VEGF. Even though several ED marker levels were higher in post-HFRS patients than in healthy individuals, most of the stated differences were not significant and do not offer sufficient evidence for significant ED in HFRS patients 14 years after the disease contraction. However, future research on different HFRS patient cohorts should further explore the trends observed in this study and could provide more details on ED events at different time points after acute HFRS.

This study has several limitations. Firstly, the analyzed sample size was relatively small, resulting in lower power of the utilized statistical tests. The sample size was limited considering the status of HFRS as an epidemic disease with a relatively small incidence in Croatia and considering a relatively low response rate of post-HFRS patients 14 years after the disease onset. Another limitation is a lack of complete data on hematological, biochemical, urine and ED markers at earlier time points after HFRS contraction, which would allow for a detailed longitudinal analysis of HFRS sequelae. Additionally, the relatively low number of recruited post-HFRS patients infected with DOBV did not allow for comparisons of renal function and ED in post-HFRS patients infected with PUUV and DOBV. Furthermore, all of the patients included in this study were recruited from a single hospital center, limiting the persuasiveness of our findings. All in all, the results presented in this study point towards mild renal dysfunction in convalescent HFRS patients 14 years after the acute disease, possibly resulting from local inflammation, hypertension and renal ED. However, more research is needed to fully understand the role of ED in chronic HFRS sequelae.

## Figures and Tables

**Table 1:** Participants’ demographic and clinical parameters.

Parameters	N (%)/Median (Range)
Patients (N = 30)	
Male sex	30 (100.0%)
Age (years)	41 (31–49)
Body weight (kg)	89 (61–111)
Systolic pressure (mmHg)	130.0 (110.0–160.0)
Diastolic pressure (mmHg)	86.7 (60.0–120.0)
Mean arterial pressure (mmHg)	101.1 (78.2–126.6)
Viral species	
PUUV	27 (90.0%)
DOBV	2 (6.7%)
Undetermined	1 (3.3%)
Symptoms	
Headache	15 (50.0%)
Depression	15 (50.0%)
Painful lumbar succussion	11 (36.7%)
Hepatomegaly	3 (10.0%)
Frequent urination	2 (6.7%)
Splenomegaly	2 (6.7%)
Healthy individuals (N = 30)	
Male sex	30 (100.0%)
Age (years)	38.8 (30–49)
Body weight (kg)	92 (73–110)
Systolic pressure (mmHg)	123.3 (100.0–150.0)
Diastolic pressure (mmHg)	80.0 (60.0–100.0)
Mean arterial pressure (mmHg)	91.6 (74.9–116.5)

PUUV = Puumala virus; DOBV = Dobrava virus.

**Table 2:** Comparison of selected hematological and biochemical parameters in post-HFRS patients and healthy individuals.

**Hematological** **Parameters**	**Reference Values**	**Post-HFRS** **Patients** **Median (Range)**	**Healthy** **Individuals** **Median (Range)**	***p*-Value**
ESR (mmh)	4–24	6 (1–31)	4 (1–12)	0.025
Erythrocytes (×10^12^/L)	4.30–5.72	4.8 (4.2–6.0)	5.1 (4.0–5.7)	0.340
Hemoglobin (g/L)	138–175	149 (130–178)	153 (118–171)	0.599
Leukocytes (×10^9^/L)	3.4–9.7	6.8 (3.9–12.2)	6.7 (4.1–9.1)	0.871
Platelets (×10^9^/L)	158–424	231 (149–492)	227 (169–344)	0.773
vWF (IU/L)	0.5–1.5	1.24 (0.64–1.80)	1.04 (0.47–1.60)	0.008
D-dimers (mg/L)	<0.5	0.2 (0.1–0.7)	0.2 (0.1–0.7)	0.917
**Biochemical** **Parameters**	**Reference Values**	**Post-HFRS** **Patients** **Median (Range)**	**Healthy** **Individuals** **Median (Range)**	***p*-Value**
AST (U/L)	1–37	22 (14–59)	25 (15–45)	0.445
Urea (mmol/L)	3.6–7.2	5.8 (2.4–7.3)	6.1 (4.1–8.4)	0.105
Uric acid (μmol/L)	142–416	319 (157–556)	297 (213–387)	0.025
T3 (mmol/L)	1.26–2.75	1.80 (1.18–2.27)	1.73 (0.95–2.66)	0.283
T4 (mmol/L)	58–161	87 (75–139)	104 (67–130)	0.005
CRP (mg/L)	0.0–5.0	2.5 (0.5–22.0)	1.2 (0.5–7.7)	0.005
IgA (g/L)	0.7–4.0	2.3 (0.9–6.4)	1.8 (0.9–3.7)	0.014

HFRS = hemorrhagic fever with renal syndrome; ESR = erythrocyte sedimentation rate; vWF = von Willebrand factor; AST = aspartate aminotransferase; T3 = triiodothyronine; T4 = thyroxin; CRP = C-reactive protein; IgA = immunoglobulin A.

**Table 3:** Comparison of 24 h urine test results in post-HFRS patients and healthy individuals.

24 h-Urine Analysis	Reference Values	Post-HFRS Patients Median (Range)	Healthy Individuals Median (Range)	*p*-Value
Sodium (mmol/d)	120–250	157 (20–503)	230 (107–362)	0.040
Potassium (mmol/d)	50–100	62 (34–129)	65 (28–94)	0.010
Chloride (mmol/d)	128–257	185 (32–459)	223 (119–407)	0.061
Urea (mmol/d)	250–570	346 (180–640)	445 (309–776)	0.112
Proteins (mg/d)	25–150	133 (51–262)	90 (13–157)	0.023
Microalbumin (mg/d)	1.0–30.0	6.9 (2.7–131.0)	5.3 (0.7–8.2)	0.011
β2-microglobulin (mg/L)	0.00–0.20	0.20 (0.08–0.33)	0.10 (0.13–23.00)	0.027

HFRS = hemorrhagic fever with renal syndrome.

**Table 4:** Comparison of first morning urine test results in post-HFRS patients and healthy individuals.

First morning Urine Analysis	Post-HFRS Patients N (%)	Healthy Individuals N (%)	*p*-Value
Positive nitrites (>0 mmol/L)	1 3.6%	0 (0.0%)	0.999
Proteins > 0.2 mg/dL	7 (23.3%)	2 (6.7%)	0.146
Erythrocytes > 2 per field of view	11 (36.7%)	3 (10.0%)	0.030
Leukocytes > 2 per field of view	17 (56.7%)	10 (33.3%)	0.119

HFRS = hemorrhagic fever with renal syndrome.

**Table 5:** Comparison of specific endothelial dysfunction markers in post-HFRS patients and healthy individuals.

**ED Markers in Serum**	**Post-HFRS** **Patients** **Median (Range)**	**Healthy** **Individuals** **Median (Range)**	***p*-Value**
P-selectin (ng/mL)	101.4 (45.6–194.2)	84.0 (24.2–311.2)	0.265
E selectin (ng/mL)	50.3 (21.7–121.9)	46.3 (31.0–103.0)	0.505
sICAM-1 (ng/mL)	205.2 (89.9–566.3)	165.3 (99.1–430.4)	0.203
sVCAM-1 (ng/mL)	439.3 (284.6–698.6)	507.9 (301.9–880.5)	0.299
VEGF (pg/mL)	278.5 (57.0–824.0)	236.0 (58.0–960.0)	0.921
**ED Markers in Urine**	**Post-HFRS** **Patients** **Median (Range)**	**Healthy** **Individuals** **Median (Range)**	***p*-Value**
P-selectin (ng/mL)	0.1 (0.0–0.4)	0.1 (0.0–1.0)	0.382
E selectin (ng/mL)	0.2 (0.0–0.4)	0.2 (0.0–0.4)	0.948
sICAM-1 (ng/mL)	0.0 (0.0–1.6)	0.0 (0.0–4.0)	0.761
sVCAM-1 (ng/mL)	20.6 (0.0–97.0)	5.3 (0.1–100.9)	0.080
VEGF (pg/mL)	276.1 (1.1–1847.0)	242.2 (117.0–778.0)	0.294

ED = endothelial dysfunction; HFRS = hemorrhagic fever with renal syndrome; sICAM-1 = soluble intercellular adhesion molecule 1; sVCAM-1 = vascular intercellular adhesion molecule 1; VEGF = vascular endothelial growth factor.

**Table 6:** Binary logistic regression model classifying post-HFRS patients and healthy individuals.

Parameter	Coefficient	Odds Ratio	*p*-Value
CRP	0.990	2.691	0.050
24 h urine microalbumin	0.338	1.474	0.048
Serum sVCAM-1	−0.026	0.974	0.043
Urine VEGF	0.015	1.015	0.041

CRP = C-reactive protein; sVCAM-1 = vascular intercellular adhesion molecule 1; VEGF = vascular endothelial growth factor.

## Data Availability

The data presented in this study are available on request from the corresponding author.
